# Remineralization and anti-demineralization effect of orthodontic adhesives on enamel surrounding orthodontic brackets: a systematic review of in vitro studies

**DOI:** 10.1186/s12903-024-05237-y

**Published:** 2024-11-28

**Authors:** Kareem Hamdi, Ahmed Elsebaai, Mostafa A. Abdelshafi, Hamdi H. Hamama

**Affiliations:** 1 Operative Dentistry Department, Faculty of Dentistry, Zagazig University, Zagazig, Egypt; 2 Pediatric Dentistry Department, Faculty of Oral and Dental Medicine, Delta University for Science and Technology, Mansoura, Egypt; 3 Dental Biomaterials Department, Faculty of Dentistry, Mansoura University, Mansoura, Egypt; 4 Conservative Dentistry Department, Faculty of Dentistry, Mansoura University, Mansoura, Egypt; 5 Kareem Dental Clinic, Al Guesh St, Mansoura City, 35516 Egypt

**Keywords:** Enamel, Remineralization, Orthodontic adhesives, White spot lesion, Demineralization, Systematic review

## Abstract

**Objective:**

White spot lesions are initial sign of enamel caries that compromise esthetic appearance following orthodontic treatment. Thus, the systematic review was conducted to evaluate the remineralization potential of orthodontic adhesives on early-enamel lesions surrounding orthodontic bracket.

**Methods:**

Search strategy was performed through three databases (PubMed, Web of Science, and Scopus). A total number of 1712 studies were identified for being potentially relevant, 62 among them were further assessed. Finally, 24 studies were included in this systematic review after adopting the eligibility criteria. The methodologies used to assess enamel remineralization were micro-computed tomography, cross-sectional microhardness, and polarized light microscopy for evaluating remineralization extent; surface microhardness, color change, and laser-induced fluorescence for evaluating superficial surface mineralization; Fourier Transform InfraRed, and Energy Dispersive Spectroscopy technique for chemical characterization.

**Results:**

Bioactive glass (BAG), nano-hydroxyapatite (n-HAP), nano-amorphous calcium phosphate (n-ACP), nano-calcium fluoride (n-CaF_2_), fluorinated amorphous calcium phosphate nanoparticles (AFCP), and ammonium-based methacrylate monomer were incorporated into orthodontic adhesives. Seven out of the 24 included studies utilized commercially available orthodontic adhesives. While the remaining studies utilized experimental bioactive adhesives; 13 studies evaluated adhesives containing (BAG), two studies evaluated adhesives containing (n-ACP), one study evaluated adhesives containing (AFCP), and one study evaluated adhesives containing (n-HAP). Orthodontic adhesives containing the previously mentioned additives showed significant remineralization power compared to control group. Majority of studies that evaluated bioactive-based orthodontic adhesives revealed significant remineralization effect in comparison with their corresponding control groups. Out of 24 studies, 8 have assessed ion-release. However, few numbers of included studies evaluated the ion-release peak values. The output of most studies reported a significant increase of ion-release over time. Only one study reported a mark decrease of calcium and phosphate ions after 72 h. Following the high risk of bias in the majority of studies, and lack of standard evaluation protocol, meta-analysis was not conducted.

**Conclusion:**

The outcome of the included studies supports the effectiveness of incorporation of remineralizing agents into orthodontic adhesives.

**Supplementary Information:**

The online version contains supplementary material available at 10.1186/s12903-024-05237-y.

## Introduction

Fixed orthodontic appliance is an integral part of the orthodontics during orthodontic treatment. Some drawbacks are associated with this line of treatment including food and plaque stagnation, difficult flossing and difficult teeth brushing and cleaning. Furthermore, the irregular surface of orthodontic brackets, and bands may limit the self-cleaning mechanism of oral musculature. All of these factors surely accelerate the rate of enamel demineralization and increase the risk of development of white spot lesions (WSLs) around orthodontic brackets [1].

WSLs are considered as the early clinical sign of enamel carious lesion that can be clinically detected with naked eye and compromise the esthetic appearance of individuals following orthodontic treatment [2]. Clinically, WSLs might develop in the 4th week after initiating orthodontic treatment, particularly, in patients with poor oral hygiene [3]. It was reported that 37% of the treated patients had at least one new post orthodontic WSL, while 24% of the treated teeth develop at least one new WSL [4]. Based on previously published meta-analysis for evaluation of WSLs, the incidence of developing new carious lesions during orthodontic treatment was 45.8%, with a prevalence rate of 68.4% in patients under orthodontic treatment [5]. A recently published evidence-based study stated that the prevalence of post orthodontic WSLs in different studies varies from 33.8 to 97%. The variation in prevalence of WSLs between the studies ascribed to method/criteria of evaluation, pre-existing enamel lesions, and patient related factors [1]. Generally speaking, the beforementioned published evidence highlight the high prevalence of post-orthodontic WSLs, necessitating both patients and caregivers to pay attention.

To reduce the likelihood of WSLs developing around orthodontic brackets, several preventive agents have been recommended during orthodontic treatment [6]. These including application of fluoride varnish [6], daily use of Casein Phosphopeptide Amorphous Calcium Phosphate CPP-ACP [3], and use of lingual orthodontic appliance [7]. A previously published systematic review reported that, such preventive measures may have short-term preventive effect, while there is no robust evidence of their effect on the long track records [8]. Furthermore, the preventive effect of these measures might be affected with patient compliance.

Orthodontic adhesives are essential for bonding orthodontic brackets and bands to the tooth substrate. The bonded interface is the weakest link which is susceptible to plaque accumulation and development of further decalcification [9]. Therefore, it is imperative to develop remineralization and anti-demineralization strategy via novel orthodontic adhesive to avoid further decalcification. After comprehensive searching the currently available published scientific literature, it was found that several laboratory studies evaluated remineralization power of different experimental orthodontic adhesives. Meanwhile, few randomized clinical trials were conducted for same purpose. Consequently, this systematic review was designed to evaluate the output of previously published laboratory studies and translate knowledge from bench to clinic by answering the focus question “Do orthodontic adhesives remineralize or prevent demineralization of enamel surrounding orthodontic brackets?”.

## Methods

The current systematic review was conducted following Preferred Reporting Items for Systematic Reviews and Meta-Analyses PRISMA Checklist [10]. The protocol was registered in the international Prospective Register of Systematic Reviews (PROSPERO) under number (CRD42024574945).

### Eligibility criteria

The current systematic review includes the experimental studies that evaluated the remineralization potential of different experimental, modified, and remineralizing orthodontic adhesives around orthodontic brackets or appliance. Generally, the inclusion criteria was based on PECOS methodology [11]: Population (P): Teeth, Exposure (E): exposure to orthodontic remineralizaing adhesives, Comparison (C): comparison with orthodontic adhesives, Outcomes (O): evaluating enamel surface / subsurface remineralization after application of orthodontic adhesive (S): In vitro and in situ studies. No data, sex or language restrictions were applied to the search strategy. The exclusion criteria includes; review studies(literature, systematic, scope, and umbrella review), letters to editor, case reports, in vivo studies, clinical trials, in vitro studies that assessed the remineralization effect of fluoride, bioactive, and biomimetic-based remineralizing agents, in vitro studies that used non-human teeth or experimental animal model, in vitro studies that assessed other properties of orthodontic adhesive rather than remineralization power on enamel substrate, and in vitro studies that didn’t adhere to ideal cariogenic challenge model (demineralization-remineralization) either chemical or biofilm model.

### Information and search strategy

An electronic search of the literature was conducted through the following databases: PubMed (including Medline), Scopus and Web of science from 2005 up to 2024. More information about search strategy in different database are presented in Supplementary file 1. All references were managed, and the duplicated hits were removed by a reference manager software (EndNote X9^®^ Basic-Thomson Reuters, New York, USA).

### Data extraction

This part was performed in two separate phases. In phase one, two independent reviewers (K.H and M.A) independently screened and evaluated the titles and abstracts of all studies that were identified in electronic database. In phase two, the same reviewers screened full text of the studies that were relevant independently, and then collected the key information from the studies. The references in those relevant studies were also checked to ensure including any interesting papers that might be missed during the search. Finaly, selection of studies was basically depending on full-text assessment. When disagreement in selection happens between the reviewers, third reviewer was invited to make a final decision in this regard.

### Data items

Specific data elements were identified from each study including demineralization technique, sample size, study duration, used orthodontic adhesive, and methods of evaluation and characterization of enamel substrate. Data was collected independently by two reviewers (K.H and M.A) after whole text assessment and presented in Table 1.

**Table 1:** Study characteristics

Refs	Demin	Sample size	Adhesives used in the study	Study duration (Repetition of cariogenic challenge cycles)	Technique used to assess remineralization/demineralization extent	Technique used for surface evaluation	Chemical surface characterization	Surface micro-morphological analysis
Choi, A. et al. [21]	Chemical	20	Self-adhesive resin cement SAR (Ortho Connect Flow; GC Corp, Tokyo, Japan) containing MPN nano particles with different conc. 0,1,3,5 wt%	14 days	Micro-CT			
Choi, A. et al. [22]	Chemical	20	Self-adhesive resin cement containing MPC and MPN with different conc. SAR SAR + 3% MPC SAR + 5% MPC SAR + 1% MBN + 3% MPC SAR + 3% MBN + 3% MPC	14 days	Micro-CT			
Iijima, M. et al. [14]	Chemical	56	1- 4-META/MMA-TBB-based fluoride containing resin adhesive (Super-Bond/F3). 2-Super-Bond (SEP resin adhesive) 3-Transbond Plus, SEP resin adhesive 4-Fuji Ortho LC, RMGIC	28 days	CSNH with nanoindentation			
Kim, Y. M. et al. [23]	Chemical	60	Transbond™ XT (TXT) Charmfil™ Flow Adhesive containing Silver or zinc doped bioactive glass BAG with different conc. 1-Charm Fill CF 2- Transbond TXT 3- CF + A0-10 4- CF + A1-10 5-CF + A1Z 5–10 6-CF + A1Z 5–15 7- CF + Z5-15	14 days	Micro CT			
Kohda, N. et al. [24]	Chemical	60	4-META/ MMA-TBB-based resin adhesive with different conc. Of BAG (0-50Wt.%)	14 days	CSNH			
Lee, S. M. et al. [25]	Chemical	63	Sterile saline (-ve control) 1-Adhesive + BAG 0.2 wt% 2-Adhesive + BAG 1 wt% 3-Adhesive + BAG@Ag1 0.2% 4-Adhesive + BAG@Ag1 1% 5-Adhesive + BAG@Zn50.2% 6-Adhesive + BAG@Zn5 1%	14 days	Micro-CT			
Liu, Yan. et al. [33]	Chemical	18	(1) TransBond XT -control TB (2) PEHB + 5% MAEDB [designated as PD] (3) PEHB + 5% MAE-DB + 40% NACP [designated as PND]	28 days	CSMH			SEM/AFM
Ma, Y. et al. [15]	Biologic	48	1-Transbond XT (TB) 2-RMGI (GC Ortho LC) 3-RMGI + MPC + DMAHDM 4-RMGI + MPC + DMAHDM + NACP.	14 days	CSMH PLM			SEM
Manfred, L. et al. [26]	Chemical	50	Transbond XT (control) Orthodontic new adhesives contained BAG in varying percentages: BAG62 BAG65 BAG81 BAG85	14 days	CSMH			
Nam, H. et al. [27]	Chemical	20	1-Charm Fill Flow (CF) 2-CF+ FGtBAG 1 Wt.% 3-CF+ FGtBAG 3 Wt.% 4-CF + FGtBAG 5 Wt.%	14 days	Micro-CT			
Nam, H. et al. [28]	Chemical	20	1-Transbond XT Low Flow (LV) 2-LV+ FGtBAG 1 Wt.% 3-LV + FGtBAG 3 Wt.% 4-LV + FGtBAG 5 Wt.%	14 days	Micro-CT			
Nascimento, P. et al. [37]	Biological	60 halves	Adhesive containing antibacterial monomer [2 (Methacryloyloxy)ethyl] trimethylammonium chloride (MADQUAT; Sigma-Aldrich) with varying conc. 0, 5, 10 wt %	5 days	CSMH			
Parihar, A. et al. [29]	Chemical	12 for SEM	1-Transbond-XT Resin Adhesive (3 M Unitek) after etching and priming 2-BAG-Bond: after etching and priming 3-BAG-Bond without primer	6 months				SEM
Paschos, E. et al. [16]	Chemical	85	1-Transbond Plus SEP and Transbond XT 2–37% phosphoric acid, Pro Seal (Reliance Orthodontic Products, Itasca, Ill) and Transbond XT 3-Clearfil Protect Bond (Kuraray Medical, Okayama, Japan) and Transbond XT 4–37% phosphoric acid and Light Bond (Reliance Orthodontic Products) 5- Ortho Conditioner and Fuji Ortho LC	30 days	Micro-CT PLM			
Rahmanpanah, S. et al. [34]	Chemical	40	1-control group (3MTM TransbondTM XT) 2-experimental composites containing 2% (HA2), 5% (HA5) and 10% (HA10) hydroxyapatite.	28 Day		SMH	EDX	SEM
Seifi, M. et al. [30]	Chemical	96	1- Experimental orthodontic composite (control). 2- Experimental orthodontic composite containing 1% nBG@Ag. 3-Experimental orthodontic composite containing 3% nBG@Ag. 4- Experimental orthodontic composite containing 5% nBG@Ag. 5- GC Ortho Connect orthodontic composite (control). 6- GC orthodontic composite containing 1% nBG@Ag. 7- GC orthodontic composite containing 3% nBG@Ag. 8- GC orthodontic composite containing 5% nBG@Ag.	14 days		SMH	EDX	SEM
Shirazi, M. et al. [17]	Chemical	60	1- control group: Transbond XT (3 M, St. Paul, MN, USA) 2- Fuji II LC RMGI (GC Corp., Tokyo, Japan) 3- Fuji II LC (GC Corp., Tokyo, Japan) containing 30% BAG particles	21 days	PLM			
Song, H. et al. [31]	Chemical	40	CharmFill Flow, Dentkist, Seoul, South Korea) containing 0, 1%, 3%, and 5% Ga-doped MBN (GaMBN)	14 days	Micro-CT			
Xu, Y. et al. [36]	Chemical	32	1-Transbond XT Primer (control) 2 − 0 wt% AFCP 3–25 wt% AFCP 4–30 wt% AFCP	21 days	Micro-CT	Digital photography		
Yi, J. et al. [35]	Chemical	30	1- Transbond XT (termed as TB control) 2- GC control 3- GC + 5% nCaF2 4- GC + 10% nCaF2 5- GC + 20% nCaF2 6- GC + 30% nCaF2 7- GC + 1% DMAHDM 8- GC + 2% DMAHDM 9- GC + 3% DMAHDM 10- GC + 4% DMAHDM 11- GC + 20% nCaF2 + 3% DMAHDM	30 days	CSMH	SMH		
Bhushan, R. et al. [18]	Chemical	80	1-Transbond XT 2-Transbond Plus color change adhesive 3-GC Fuji Ortho LC 4- Vitremer	14 days				SEM
Demircioglu, R. et al. [19]	Biological	60	1-Transbond XT Primer + Transbond XT Light Cure Adhesive (3 M Unitek, Monrovia, CA, USA), 2-GC Ortho Connect Light Cure Adhesive (GC Crop, Tokyo, Japan) 3- Transbond™ Plus Self Etching Primer + Transbond XT Light Cure Adhesive (3 M Unitek, Monrovia, CA, USA)	28 days		DIAGNODent pen		
Chow, C. et al. [20]	Chemical	40	(1) Transbond XT (3 M Unitek, Monrovia, CA), a light cure composite without fluoride or ACP. (2) Quick Cure (Reliance Orthodontic Products, Itasca, IL), a light cure composite with fluoride. (3) Aegis Ortho (Bosworth Co., Skokie, IL), a light cure composite with 38% ACP fillers	28 days for XPS 42 days for PLM	PLM		X-Ray Photoelectron Spectrophotometry (XPS)	
Firoz, H. [32]	Chemical		1-F-BGC-1: Fluoride bioactive glass ceramic + Transbond XT B. 2- BGC-1: Bioactive glass ceramic + Transbond XT C. 3- F-BGC-2 Fluoride bioactive glass ceramic + Transbond XT 4- BGC-2 Bioactive glass ceramic + Transbond XT. 5- Control Transbond XT only	14 days			FTIR	SEM

**Abbreviations**: MPC; 2-methacryloyloxyethyl phosphorylcholine. nHAP: nano-hydroxy appetite. nACP: nano-hydroxyapatite. CSMH: Cross sectional microhardness. CSNH: cross sectional nano hardness. Micro-CT: micro-computed tomography PLM: Polarized light microscope SEM: Scan Electron Microscope. XRD: X-Ray Diffraction EDX: Energy Dispersing X-ray AFM: Atomic Force Microscope. nCaF2: nano Calcium-flouride. BAG: Bioactive Glass. AFCP: Amorphous Fluorinated Calcium Phosphate nanoparticles. (MAE-DB): 2-methacryloxylethyl dodecyl methyl ammonium bromide. MPN: Mesoporous Bioactive glass nanoparticles. DMAHDM: di-methylamino hexadecyl methacrylate. nBG@Ag: nano-silver containing bioactive glass. FGtBAG: fluorinated bioactive glass nanoparticles. PEHB: orthodontic experimental adhesive containing [PMGDM, EBPADMA, HEMA, Bis-GMA, BAPO]. PD: PEHB + 5% MAEDB. PND: PEHB + 5% MAE-DB + 40% NACP. SAR: Self -adhesive resin cement, META/ MMA-TBB: 4-Acryloyloxyethyl trimellitate anhydride/methyl methacrylate-tri-n-butylborane. SEP: self-etch primer. MBN: Mesoporous Bioactive glass Nanoparticles. F-BGC: Flouridated bioglass ceramic based adhesive. GaMBN: Gallium-doped bioactive glass nanoparticles. A0: 58-SiO, 33-CaO, 9-P2O5. A1: 58-SiO, 32-CaO, 9-P2O5, 1-Ag2O. A1Z5: 58-SiO, 27-CaO, 9-P2O5, 1-Ag2O, 5-ZnO. Z5: 58-SiO, 28-CaO, 9-P2O5, 5-ZnO

### Quality assessment of included studies

The selected studies were assessed following JBI & CRIS (Checklist for reporting laboratory studies) guidelines. Regarding methodology and treatment objective we have formulated the following parameters to assess remineralization extent of different orthodontic adhesives. Use of control group, standardization of the teeth, method of sample size calculation, estimated meaningful difference, random distribution of samples, detailed explanation about sample preparation, using multiple evaluation techniques / characterization approaches, samples prepared by single operator, observer blinding, appropriate reporting of statistical analysis, and laboratory study stimulating in vivo situations. Risk of bias assessment was performed by two reviewers (K.H and M.A) who assessed the eligible studies following the beforementioned parameters. Two reviewers (K.H and M.A) had independently scored each item as “yes”, “no” or “unclear” then classified the quality of each included study as “high”, “low” or “moderate” risk of bias. The final score of each article was determined based on calculating the percentage of “yes” answer in the pre-formulated 11 question. Therefore, the high risk of bias study has score ≤ 49%, moderate risk of bias study has score ranging of 50–69%, and the low-risk study has score ≥ 70% [12, 13]. In case of uncertainty or conflict in assessment, third reviewer was invited for judgmental decision.

### Effect measures

The results were interpreted in terms of structural, optical, mechanical, and chemical properties of treated enamel substrate as quantifiable markers of lesion depth.

### Synthesis method

Quantitative synthesis and a meta-analysis would be conducted if the included studies are sufficiently homogenous. Data items on the included studies (demineralization technique, sample size, study duration, used orthodontic adhesive, and methods of evaluation and characterization of enamel substrate) were tabulated. Findings from the studies assessing the remineralization and ion releasing outcomes were summarized separately and tabulated.

## Results

**Fig. 1:**
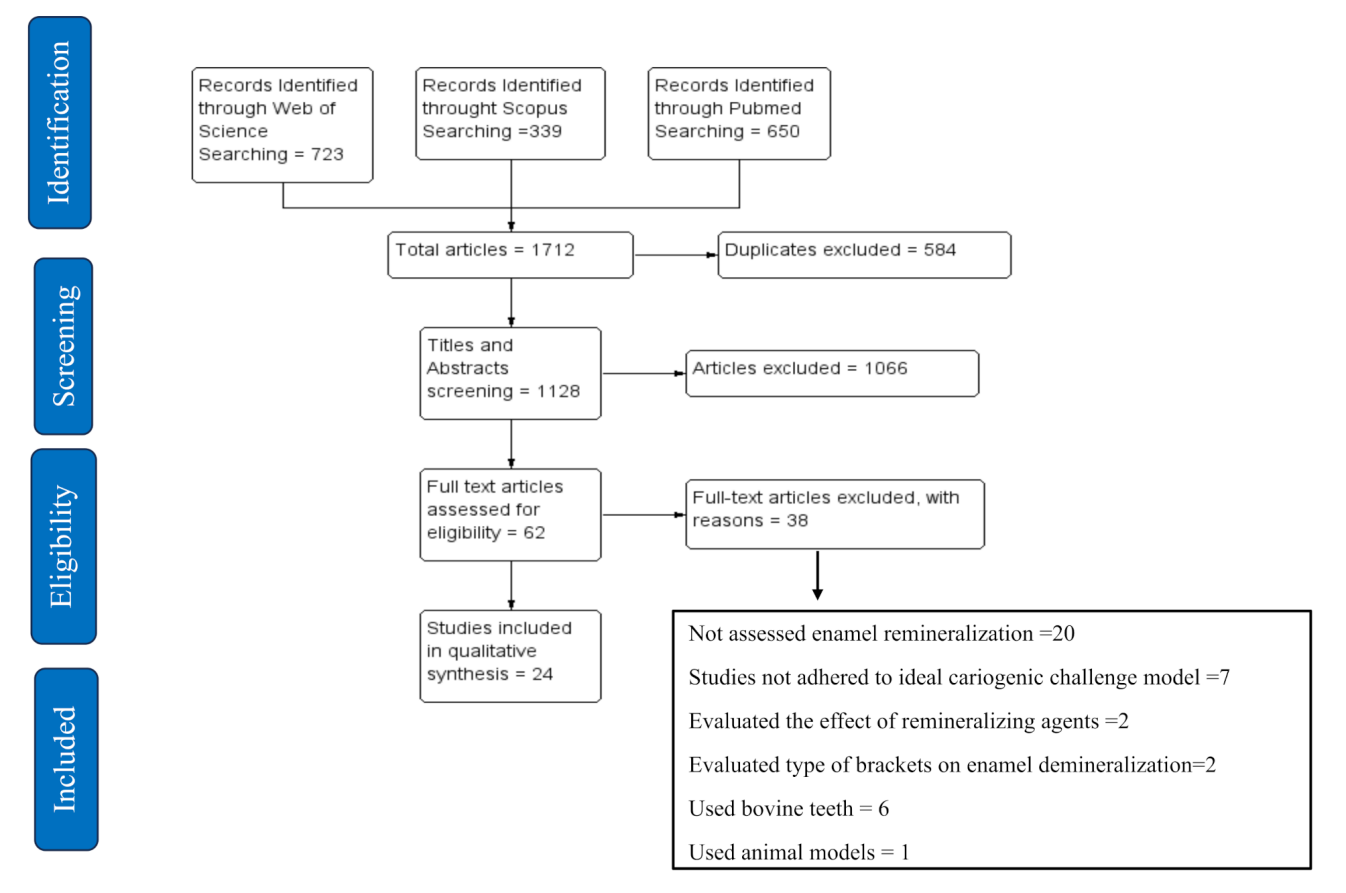
Flow diagram of the study screening and selection

After comprehensive searching the databases of PubMed, Web of Science, and Scopus, 1712 studies were initially identified for being potentially relevant. Then 1,128 studies had remained for title and abstract screening following removal of duplicates. Considering deploying the inclusion and exclusion criteria, 1.066 studies were excluded, while 62 studies were required full text checking. Finally, a total number of 24 studies were included in this systematic review (Fig. 1).

### Risk of bias assessment

**Fig. 2:**
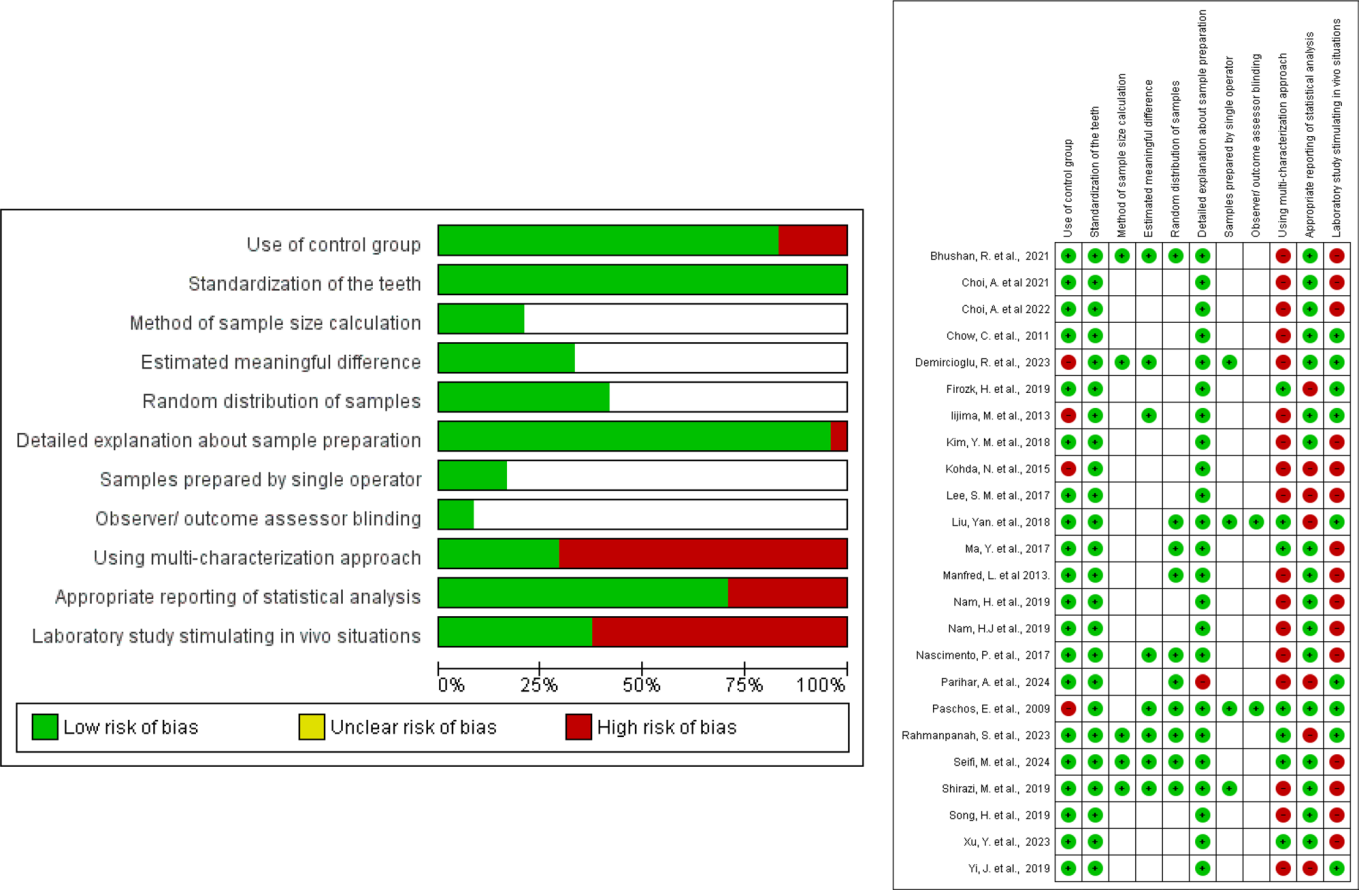
(**A**)- Risk of bias graph (**B**)-Risk of Bias Summary

According to the selected tool for risk of bias assessment, 15 studies were categorized as having high, 4 having moderate, and 5 having low risk of bias. The main methodological bias was ascribed to lack of sample size calculation, outcome assessor blinding, using single evaluation technique and several in vitro studies didn’t simulate in vivo situations. Risk of bias assessment was detailed in Table 2; Fig. 2.

**Table 2:** Risk of bias assessment

Refs	Use of control group	Standardization of the teeth	Method of sample size calculation	Estimated meaningful difference	Random distribution of samples	Detailed explanation about sample preparation	Samples prepared by single operator	Observer/ outcome assessor blinding	Using multi -characterization approach	Appropriate reporting of statistical analysis	Laboratory study stimulating in vivo situations	Risk of Bias
Choi, A. et al. [21]	Yes	Yes	Unclear	Unclear	Unclear	Yes	Unclear	unclear	No	Yes	No	High
Choi, A. et al. [22]	Yes	Yes	Unclear	Unclear	Unclear	Yes	Unclear	unclear	No	Yes	No	High
Iijima, M. et al. [14]	No	Yes	Unclear	Yes	Unclear	Yes	Unclear	Unclear	No	Yes	Yes	High
Kim, Y. M. et al. [23]	Yes	Yes	Unclear	Unclear	Unclear	Yes	Unclear	Unclear	No	Yes	No	High
Kohda, N. et al. [24]	No	Yes	Unclear	Unclear	Unclear	Yes	Unclear	Unclear	No	No	No	High
Lee, S. M. et al. [25]	Yes	Yes	Unclear	Unclear	Unclear	Yes	Unclear	Unclear	No	No	No	High
Liu, Yan. et al. [33]	Yes	Yes	Unclear	Unclear	Yes	Yes	Yes	Yes	Yes	No	Yes	Low
Ma, Y. et al. [15]	Yes	Yes	Unclear	Unclear	Yes	Yes	Unclear	Unclear	Yes	Yes	No	Moderate
Manfred, L. et al. [26]	Yes	Yes	Unclear	Unclear	Yes	Yes	Unclear	Unclear	No	Yes	No	High
Nam, H. et al. [27]	Yes	Yes	Unclear	Unclear	Unclear	Yes	Unclear	Unclear	No	Yes	No	High
Nam, H. et al. [28]	Yes	Yes	Unclear	Unclear	Unclear	Yes	Unclear	Unclear	No	Yes	No	High
Nascimento, P. et al. [37]	Yes	Yes	Unclear	Yes	Yes	Yes	Unclear	Unclear	No	Yes	No	Moderate
Parihar, A. et al. [29]	Yes	Yes	Unclear	Unclear	Yes	NO	Unclear	Unclear	No	No	Yes	High
Paschos, E. et al. [16]	No	Yes	Unclear	Yes	Yes	Yes	Yes	Yes	Yes	Yes	Yes	Low
Rahmanpanah, S. et al. [34]	Yes	Yes	Yes	Yes	Yes	Yes	Unclear	Unclear	Yes	No	Yes	Low
Seifi, M. et al. [30]	Yes	Yes	Yes	Yes	Yes	Yes	Unclear	Unclear	Yes	Yes	No	Low
Shirazi, M. et al. [17]	Yes	Yes	Yes	Yes	Yes	Yes	Yes	Unclear	No	Yes	No	Low
Song, H. et al. [31]	Yes	Yes	Unclear	Unclear	Unclear	Yes	Unclear	Unclear	No	Yes	No	High
Xu, Y. et al. [36]	Yes	Yes	Unclear	Unclear	Unclear	Yes	Unclear	Unclear	Yes	Yes	No	High
Yi, J. et al. [35]	Yes	Yes	Unclear	Unclear	Unclear	Yes	Unclear	Unclear	No	No	Yes	High
Bhushan, R. et al. [18]	Yes	Yes	Yes	Yes	Yes	Yes	Unclear	Unclear	No	Yes	No	Moderate
Demircioglu, R. et al. [19]	No	Yes	Yes	Yes	Unclear	Yes	Yes	Unclear	No	Yes	Yes	Moderate
Chow, C. et al. [20]	Yes	Yes	Unclear	Unclear	Unclear	Yes	Unclear	Unclear	No	Yes	Yes	High
Firoz, H. [32]	Yes	Yes	Unclear	Unclear	Unclear	Yes	Unclear	Unclear	Yes	No	Yes	High

### Study characteristics

All study characteristics were reported in detail in Table 1. There were no studies published before 2005. Among the relevant studies, 7 studies evaluated the remineralization potential of commercial orthodontic adhesives [14–20], while the remaining studies evaluated the remineralization potential of experimental/modified adhesives. Regarding experimental adhesives, most of the published literature evaluated the remineralization potential of orthodontic adhesives containing bioactive materials like Bioactive glass, hydroxyapatite, and amorphous calcium phosphate particles/nanoparticles. Thirteen studies evaluated the remineralization power of bioactive glass particles/ nanoparticles containing orthodontic adhesives [17, 21–32]. Some studies incorporated additional elements to the bioactive glass such as silver (Ag) [25, 30], Zinc (Zn) [25], and fluoride (F) [27, 28]. Two studies used adhesives containing amorphous calcium phosphate nanoparticles (n-ACP) [15, 33], one study used orthodontic adhesives containing hydroxyapatite nanoparticles(n-HA) [34], one study used adhesive containing nano calcium fluoride (nCaF_2_) [35], one study used adhesives containing fluorinated amorphous calcium phosphate nanoparticles (n-AFCP) [36]. Additionally, one study used orthodontic adhesives containing ammonium based methacrylate [37].

Four studies evaluated the remineralization extent of different combination incorporating biomaterials including; Mesoporous bioactive nanoparticles (MBN) + 2-methacryloyloxyethyl phosphorylcholine (MPC) [22], orthodontic experimental adhesive containing [PMGDM, EBPADMA, HEMA, Bis-GMA, BAPO] (PEHB) + 2-methacryloxylethyl dodecyl methyl ammonium bromide (MAE-DB) + nano amorphous calcium phosphate (n-ACP) [33], 2-methacryloyloxyethyl phosphorylcholine (MPC) + di-methylamino hexadecyl methacrylate (DMAHDM) + nano amorphous calcium phosphate (n-ACP) [15]. Calcium fluoride nanoparticles (nCaF2) + di-methylamino hexadecyl methacrylate (DMAHDM) [35].

### Cariogenic challenge

Chemical method for cariogenic challenge is considered the most widely used protocol [38]. The majority of studies used chemical protocol of demineralization. The typical demineralizing solution that was used in majority of studies contain calcium and phosphate ions in the mM concentration range in a 0.05 or 0.075 M acetate buffer. The final pH was sometimes adjusted with KOH to be ranged from 4.3 to 5. Only, 2 studies used lactic acid mixed with polymer (carboxymethylcellusolse) to obtain acidic gel [33, 35].

Regarding biological protocol, 3 studies used biologic/biofilm model for cariogenic challenge [15, 19, 37]. Cariogenic challenge by biologic model simply depends on specimens’ incubation with *Streptococcus mutans*, or mixture of *Streptococcus mutans* and *Lactobacillus*, or mixture of 6 different cariogenic bacteria in growth media which usually supplied with sucrose [38]. Incubation time range from 24 h to 5 days. In the current systematic review, 2 studies used saliva collected from healthy volunteers without active carious lesions or soft tissue inflammation. Volunteers were abstained from food and drink intake for about 2 h [15, 37]. The culture media in these studies was mucin supplied with sucrose. While, one study used culture of *Streptococcus mutans* cultivated in brain heart infusion BHI that was supplemented with sucrose solution [19].

### Methodologies and assessment techniques

Different techniques have been encountered evaluating the remineralization potential of used remineralizing adhesives. These techniques are varied from techniques evaluating remineralization extent or subsurface extent, techniques evaluating superficial surface remineralization, techniques evaluating mineral / elemental content, and techniques for micromorphological characterization.

#### Evaluation of remineralization extent / subsurface remineralization

Thirteen studies evaluated the remineralization extent or subsurface remineralization using spatial information either by micro computed tomography (micro-CT) [16, 21–23, 25, 27, 28, 31, 36] or using polarized light microscope PLM [15–17, 20]. One common study between them used both techniques [16]. Regarding indirect evaluation of remineralization extent, 7 studies used cross-sectional hardness technique for evaluation remineralization extent [14, 15, 24, 26, 33, 35, 37].

#### Surface remineralization

Superficial surface remineralization was assessed by different protocols including surface hardness, color change, and laser fluorescence. Three studies used surface hardness for evaluating superficial surface mineralization [30, 34, 35], while single study used laser fluorescence technology “DIAGNOdent” [19], and another single study used digital photography [36].

#### Chemical characterization / elemental analysis

From the relevant studies, one study used Fourier Transform InfraRed FTIR technique [32], two studies used Energy Dispersive Spectroscopy EDS for chemical characterization [30, 34], and finally, single study used X-Ray Photoelectron Spectroscopy XPS for chemical characterization of treated enamel [20].

#### Surface micromorphological analysis

In addition to remineralization extent, surface mineralization, and chemical characterization, 7 studies evaluated the micromorphological structure of the treated enamel. All of 7 studies used SEM [15, 18, 29, 30, 32–34], which is a qualitative approach that can illustrate abrasive and erosive changes, or mineral depositions over treated enamel substrate [39]. While single study used Atomic Force Microscope AFM to confirm the SEM findings [33].

### Remineralization results (primary outcome)

Seven studies used cross-sectional hardness in GPa to evaluate the remineralization extent of included orthodontic remineralizing adhesives [14, 15, 24, 26, 33, 35, 37]. There was a variation between the relevant studies according to duration or repetition of pH cycling. Two studies used 28-day cycles [14, 33], three studies used 14-day cycles [15, 24, 26], one study used 30-day cycles [35], and one study used 5-day cycles for pH cycling [37]. All of 7 studies showed significant remineralization extension of remineralizing orthodontic adhesives under and around orthodontic brackets. As beforementioned, nine studies used micro-CT technique for remineralization extent/ lesion depth assessment in µm. The nine studies showed significant remineralization extent of remineralizing orthodontic adhesives [16, 21–23, 25, 27, 28, 31, 36]. Among them, one study used 30-day pH cycling [16], and one study used 21-day pH cycling [36], while the rest of nine studies used 14-day pH cycling. Furthermore, the four studies used polarized light microscope for assessment of remineralization extent / lesion depth in µm showed significant reduction of lesion depth of tested remineralizing orthodontic adhesives [15–17, 20]. Out of three studies used surface hardness in GPa to assess superficial enamel surface mineralization, two showed significant remineralization effect of remineralizing orthodontic adhesive [34, 35], while one study showed that there was no significant difference between control group and experimental adhesives used BAG in different concentration [30]. Additionally, one study used DIAGNODent laser fluorescence in numerical values showed significant difference between evaluated orthodontic adhesives [19], and one study that used digital photography in ΔE showed less color change of enamel treated with n-AFCP adhesives compared to control one [36]. Regarding chemical characterization using FTIR in characteristic peaks single study reported characteristic remineralization of the treated enamel substrate [32], however, this study didn’t report definitive statistical values. Out of two studies used EDS in characteristic peaks or Ca/P ratio, one showed increase of calcium and phosphate peaks in the experimental adhesives in comparison to control adhesives [34]. Furthermore, single study used XPS for elemental analysis showed significant difference between tested orthodontic adhesives [20].

As beforementioned, 7 studies used SEM for micromorphological analysis [15, 18, 29, 30, 32–34], 2 out of 7 studies used SEM evaluation technique alone [18, 29], while the remaining studies used SEM as an adjunctive or confirmatory technique. Among these two studies, one study showed statistically significant difference between the tested orthodontic adhesives [18], while the other study didn’t report statistical analysis in this regard [29].

Regarding the experimental adhesives, out of 13 studies evaluated bioactive glass particles/nano nanoparticles, 9 reported definitive statistical significant values in remineralization potential of these adhesives compared to other control orthodontic adhesives [17, 21–24, 26–28, 31]. Single study showed no statistically significant difference between experimental orthodontic adhesives and control groups regarding surface hardness [30], meanwhile there were three studies that didn’t report definitive statistical values [25, 29, 32]. Additionally, studies that used orthodontic adhesives containing n-HAP, n-ACP, n-AFCP, and n-CaF2 particles showed statistical significant difference compared to control orthodontic adhesives [15, 33–36]. Furthermore, studies that used orthodontic adhesives containing ammonium based methacrylate showed significant remineralization power compared to control adhesives [37].

Regarding commercial orthodontic adhesives, two studies reported significant remineralization power of Fuji Ortho LC [14, 16], two studies reported significant remineralization potential of Transbond Plus color change adhesive and Transbond™ Plus Self Etching Primer + Transbond XT Light Cure Adhesive [18, 19], and one study reported significant remineralization power of Quick Cure orthodontic adhesive [20]. Remineralization results were presented in supplementary file.2.

### Ion-release results (secondary outcome)

Eight studies from the relevant studies evaluated the ion-release form the tested orthodontic adhesives [14, 23, 24, 27, 28, 31, 33, 35], four of relevant studies evaluated fluoride release [14, 27, 28, 35], two evaluated Calcium and phosphate ions release [23, 33], while one study evaluated phosphate ion-release only [31], and another study evaluated calcium ion-release beside other minor elements [24].

Regarding fluoride release, two studies reported significant fluoride release of the following orthodontic adhesives; Fuji Ortho LC up to 6-months [14], and Orthodontic adhesives containing nCaF_2_ up to 56 days [35]. Regarding Ca and P ions release, one study used experimental adhesive quaternary ammonium resin monomer + 40% n-ACP reported significant increase of Ca and P ions up to 42 days compared to control group [33]. Additionally, another study reported significant increase of P ion-release with increasing Gallium-doped bioactive glass nanoparticles GaMBN up to 14 day compared to control group [31]. Ion-release results were presented in supplementary file. 3.

## Discussion

Several remineralizing and anti-bacterial components have been incorporated into orthodontic adhesive to maximize its therapeutic and remineralizing effect around orthodontic brackets. After search in published literature, there were several laboratory studies have been published, while few randomized clinical trials have been published from 2005 in this topic [40–45]. Therefore, the aim of the current systematic review is to summarize the results of included laboratory studies to help the dental scientific community to design a high-quality in vitro study, then translate knowledge from bench to clinic. Remineralization of enamel lesions requires the presence of partially demineralized apatite crystals that can grow to their original size after being exposed to solution supersaturated with respect to apatite. Therefore, surface remineralization of carious lesions developed during orthodontic treatment is common, leaving the body of the lesion as a white scar under a shiny hard surface. The remineralized surface layer of the lesion protects the underlying lesion body not only from demineralization, but also from further remineralization. On rare occasions, the lesion body may be remineralized when the surface layer over it has been lost and plaque in the rough area is controlled. In such conditions there is free access for considerable salivary calcium, phosphate and fluoride ions [46]. Yu, O.Y, et al. [47], stated that the cariogenic challenge used for in vitro studies can be divided into biofilm models and chemical models. The biofilm models can be further divided into closed system models and open system models. Closed models usually utilize well cell culture plates to incubate the cariogenic bacteria together with the enamel or dentin substrate., meanwhile, open system models simulate oral environment via utilizing oral biofilm reactor or artificial mouth model. Regarding chemical models, they can be further divided into simple mineralization models and pH-cycling models. Simple mineralization model aims to use mild acids that can cut dawn the remineralization process to create demineralized lesion. While, pH cycling model is based on a scheme in which a pH neutral environment was periodically interrupted by acid challenges to mimic periodic alternation of pH [47]. In light of beforementioned, several studies have been founded in published literature assessing the remineralization / caries prevention potential of orthodontic adhesives, Neither the less, these studies were excluded from the current review as they didn’t adhere to the ideal cariogenic challenge or didn’t present clear remineralization protocol [48–54]. The current systematic review includes 24 in vitro studies that assessed the remineralization potential of different experimental and commercial orthodontic adhesives. The majority of relevant studies (up to 60%) incorporated bioactive materials as BAG, n-HAP, and n-ACP in the orthodontic adhesives. Out of 24 studies, 13 evaluated the remineralization power of bioactive glass particle/ nanoparticles containing orthodontic adhesives which represent about 50% of relevant articles. As beforementioned, out of 13 studies, 9 showed promising remineralization effect of orthodontic adhesive containing BAG. High solubility of bioactive glass was considered as a main factor that responsible for its bioactivity and biomineralization effect [55]. Rationale behind BAG remineralization is ascribed to its ability to react with saliva inducing Ca^2+^, PO_4_^3−^, and Si^4+^ release at the glass surface forming silica-rich layer [56, 57]. Such a layer act as a nucleation site for Ca and P ions, so when calcium and phosphate ions deposition continue, this layer will be crystallized into hydroxyapatite [55, 57].

Traditionally, BAG was developed by melt-quenching method, but BAG has large particle size, so it was very difficult to incorporate BAG particles into restorative or adhesive materials [57]. Accordingly, sol-gel method has been developed overcoming the large particle size of BAG by reducing its size, increasing its surface area, and making it more porous and reactive [57, 58]. In this regard, all included studies that used experimental adhesives containing BAG prepared BAG by sol-gel method, while single study prepared BAG by melting method [24]. However, majority of the studies that evaluated orthodontic adhesives containing BAG particles/nano-particles reported promising results, several concerns have been raised after reviewing most of them thoroughly. Nam, H. et al. [28], Song, H-K. et al. [31], Lee, S. et al. [25] reported significant remineralization power of BAG-based orthodontic adhesives compared to control groups, while, micro-CT images that were presented in their study didn’t depict that. Additionally, Parihar, A. et al. [29] used SEM alone in their study which has major limitations that will be mentioned thereafter. Although Firzok, H. et al. [32] reported that incorporation of bioactive glass nanoparticles in orthodontic adhesive reduces the risk of development of WSLs, a major concern has been raised as the study presented the results in figures only without reporting definitive statistical values. Additionally, Lee. S.M, et al. [25] reported remineralization results in graphs rather than definitive statistical values. In this regard, these studies were classified as having high risk of bias.

Included studies that assessed orthodontic adhesives containing n-ACP, AFCP, and n-HAP particles also reported significant remineralization effect [15, 33, 34, 36]. The remarkable remineralization effect of n-HAP, AFCP, and n-ACP is ascribed to their higher surface area, nano particle size, and superior penetration into deep demineralized zones [59]. Included studies that used SEM for micromorphological analysis of enamel treated with adhesive containing either n-ACP or n-HAP showed enamel with shallow demineralization, less depressions, and having marked granular zones [15, 34]. Only one commercially available bioactive-based orthodontic adhesive (Aegis Ortho) was evaluated, while four fluoride based orthodontic adhesive (Super Bond /F3, FUJI Ortho LC, Quick Cure, Transbond XT Transbond Plus self-etching primer TB) were evaluated.

Enamel-demineralization behavior is strongly related to the buffer capacity of ions released from materials. Therefore, ions released from the orthodontic adhesive resin specimens and their buffer capacity were evaluated and considered as a secondary outcome. Out of 24 studies, 8 evaluated ion-release of orthodontic adhesives. There are two major concerns with these findings that must be highlighted. First of them is ascribed to short duration of the ion-release test. One study evaluated ion-release after 14 days [31], and two studies evaluated ion-release after 20 days [27, 28]. Consequently, it seems logically that these studies cannot reflect the long term anti-demineralization effect of orthodontic adhesive.

Regarding the second concern, most of studies used prepared resin discs with specific dimensions (height and diameter) that were immersed either in distilled water or simulated body fluid. Discs’ size and dimension are totally different from the size and dimensions of resin that used intra-orally. Hence, this may provide overestimation or underestimation of ions release when compared to clinical situation with exposure to different pH and thermal changes.

In addition to 8 studies that evaluated the ion-release potential of orthodontic adhesives, several cited studies evaluated the anti-demineralization or preventive effect of orthodontic adhesive which may draw a different conclusion. Paschos et al. [16] stated that Fuji Ortho LC showed significant less mineral loss compared to other orthodontic adhesive due to fluoride releasing potential. Bhushan, R. et al. [18] claimed that Transbond Plus color change adhesive group was more potential in inhibition of demineralization areas compared to GC Fuji Ortho LC group and Vitremer group. Additionally, Demircioglu, et al. [19] showed similar findings as they claimed that Transbond™ Plus Self Etching Primer + Transbond XT Light Cure Adhesive showed significant reduction in demineralization compared to Transbond XT Primer + Transbond XT Light Cure Adhesive and GC Ortho LC.

There was a diversity in techniques that have been used in assessment of enamel remineralization. This systematic review classified techniques evaluated enamel remineralization into; techniques evaluated remineralization extent, techniques evaluated superficial surface mineralization, techniques evaluated chemical characterization, and techniques evaluated surface micromorphological analysis. Evaluation of remineralization extent should include quantitative (i.e. how much) and spatial (i.e. Where remineralization happened) information [38]. Spatial information can be obtained by 3D-imaging techniques as micro computed tomography (micro-CT), Optical coherence tomography (OP-OCT), and confocal laser scanning microscope (CLSM) technique [38]. Alternatively, spatial information can be obtained by 2D imaging techniques as transverse micro radiography (TMR), and polarized light microscope (PLM) which are performed over transverse sections. In contrast, remineralization extent can be assessed indirectly with cross-sectional hardness test [38]. The mechanical properties of enamel are associated with mineral content in the enamel, so cross-sectional hardness test is assumed to be suitable for measurement of surface resistance to plastic deformation by using diamond shape sharp indenters at different points from external enamel surface [60].

At level of evaluation of remineralization extent, micro-CT is more preferrable approach that providing 3D image and has advantage of being non-destructive approach compared to corresponding CLSM, and TMR. Additionally, it is more reliable approach that can map the homogeneity of remineralization a long whole enamel substrate compared to cross sectional hardness test [38, 61].

In addition to surface hardness, superficial surface mineralization was assessed in the included studies by two different techniques. Laser fluorescence using DIAGNOdent^®^ which emit red light that can be absorbed by enamel, then the re-emitted red fluorescence from enamel will be converted to numerical value according to manufacturer scale [62]. Additionally, surface color change can be measured by digital photography followed by image analysis using specific software to detect color changes. Consequently, red, green, and blue values of the enamel surface are converted to CIE Lab color space (L* lightness, a* redness, b* yellowness). The color difference (ΔE) between baseline or demineralized and remineralized points are calculated using the following formula: ΔE = [(ΔL*)2 + (Δa*)2 + (Δb*)2 ] [36]. It worth pointing out that, both techniques cannot ascertain remineralization extent or determine the lesion depth. Consequently, it is recommended to be combined with other spatial information 3D or 2D imaging techniques.

Hydroxyapatite HA is the unit structure of dental enamel which consists of calcium and phosphate mineral phase. X-ray diffraction XRD can identify this mineral phase in remineralized enamel [63]. When x-ray beam hit an atom, it will be diffracted in different directions, so when several atoms are present the diffracted beam can combine in additive manner. Furthermore, the chemical structure of the atoms can affect the intensity of recorded beam. Additionally, the width of recorded beams usually gives information about crystallinity of the examined material or substrate [64]. Fourier Transform InfraRed (FTIR) and Raman spectroscopy (RS) are vibrational frequency-based techniques that considered reliable and give sufficient information about mineral content of enamel substrate. FTIR basically depends on absorption of light with different energy, then the absorbed light will be converted into vibrational energy representing the molecular fingerprint of the sample. Each molecule or chemical structure has its a unique spectral fingerprint, making FTIR analysis a reliable tool for chemical characterization [65]. While, Raman spectroscopy utilizes a laser beam which will hit the atom producing vibrational frequencies. Most of these vibration peaks are related to phosphate and carbonate groups which will reflect the remineralization extent [65]. Weight or atomic ratio of mineral phase can be further detected by using Energy Dispersive Spectroscopy EDS, as EDS detector collects the emitted X-Ray light from atoms and quantifying the mineral content [66].

At level of chemical characterization, XRD technique is highly recommended for enamel surface characterization compared to other chemical characteristic techniques. It is described in dental literature as is an unambiguous signature of the chemical nature of the mineral phase that can also provide sufficient information about crystallinity [38, 67, 68]. Alternatively, data obtained from vibrational frequency-based techniques like FTIR and Raman spectroscopy usually poses difficult interpretation. In contrast, a lot of concerns have been raised about EDS, as the technique has high sensitivity to surface topology, therefore the degree of surface roughness may affect the result [38]. Accordingly, the technique is not recommended to be used alone, it must combined or confirmed with other chemical characterization technique.

To end up this section, assessment of remineralization extent using micro-CT combined with XRD and / or Raman spectroscopy / FTIR seems to be suitable evaluation technique to be utilized in future upcoming research. Beside remineralization extent, and chemical characterization, surface micromorphological analysis is highly recommended using AFM. AFM is a powerful tool that can deliver a high-resolution image and quantify the subtle changes in enamel compared to SEM [39].

Regarding quality assessment of the relevant studies, the majority of studies, 15 study, were reported to have high risk of bias due to methodological errors. Accordingly, the overall strength of the current evidence is considered weak. Out of 24, studies, 19 studies didn’t report sample size calculation. Limited sample size usually reduce the power of study, loss the estimated meaningful difference, and increase the risk of false negative results (type II error) [69]. Fourteen studies were considered not simulating clinical situation due to short pH cycling period. Several published studies considered the period of 4 weeks is sufficient for enamel mineralization [70, 71]. Meanwhile, another published evidence reported that the period of 23 days seems to be enough for enamel remineralization [72]. Therefore, we considered studies that used period less than 23 days didn’t simulate the clinical conditions. Most of the studies neither reported blinding in samples preparation nor investigations which surely carries a great risk of bias. Furthermore, enamel surface evaluation using single technique, particularly, surface micromorphological analysis is considered highly biased, non-reliable protocol which was reported in several studies [18, 29, 50]. After qualitative analysis of the included studies, it was not possible to conduct a quantitative analysis due to methodological heterogenicity between the included studies.

## Limitation

At the level of the study, it is essential to acknowledge the major limitations of the current systematic review, which should be considered in the upcoming research. The primary limitation is ascribed to the high risk of bias of many included studies. Additionally, there is distinguished heterogeneity among the reviewed studies in enamel remineralization evaluation methods. The secondary limitation is ascribed to few numbers of included studies which assessed ion-release of used orthodontic adhesives. Consequently, prevention of secondary caries around orthodontic brackets was not properly assessed.

## Conclusion

According to the findings of the current systematic review, incorporation of remineralizing agents into orthodontic adhesives showed promising remineralization and therapeutic effect. Additionally, the fluoride-releasing ability of the composite resin adhesive and RMGIC adhesive prevents enamel demineralization and the deterioration in mechanical properties of enamel around brackets. Neither the less, the current systematic review is considered weak evidence due to heterogeneity and high risk of bias of most included studies. Furthermore, ion-release property was not properly assessed due to limited number of included studies and heterogenicity in between them. For more homogenous studies with low risk of bias, sample size calculation, standard evaluation protocol (as beforementioned), blinding in sample preparation/ investigation and proper simulation of clinical conditions must be followed in upcoming research.

## Electronic supplementary material

Below is the link to the electronic supplementary material.

Supplementary Material 1

Supplementary Material 2

Supplementary Material 3

Supplementary Material 4

## Data Availability

All the data presented during the study are included in the article.

## Abbreviations

WSLs: White spot lesions
MPC: 2-methacryloyloxyethyl phosphorylcholine
nHAP: nano-hydroxy appetite
nACP: nano-hydroxyapatite
CSMH: Cross sectional microhardness
CSNH: Cross sectional nano hardness
Micro-CT: Micro-computed tomography
PLM: Polarized light microscope
SEM: Scan Electron Microscope
XRD: X-Ray Diffraction
EDX: Energy Dispersing X-ray
AFM: Atomic Force Microscope
nCaF2: Nano Calcium-flouride
BAG: Bioactive Glass
AFCP: Amorphous Fluorinated Calcium Phosphate nanoparticles
(MAE-DB): 2-methacryloxylethyl dodecyl methyl ammonium bromide
MPN: Mesoporous Bioactive glass nanoparticles
DMAHDM: di-methylamino hexadecyl methacrylate
nBG@Ag: Nano-silver containing bioactive glass
FGtBAG: Fluorinated bioactive glass nanoparticles
PEHB: Orthodontic experimental adhesive containing [PMGDM, EBPADMA, HEMA, Bis-GMA, BAPO
PD: PEHB + 5% MAEDB
PND: PEHB + 5% MAE-DB + 40% NACP
SAR: Self -adhesive resin cement
META/MMA-TBB: 4-Acryloyloxyethyl trimellitate anhydride/methyl methacrylate-tri-n-butylborane
SEP: Self-etch primer
MBN: Mesoporous Bioactive glass Nanoparticles
F-BGC: Flouridated bioglass ceramic based adhesive
GaMBN: Gallium-doped bioactive glass nanoparticles
A0: 58-SiO, 33-CaO, 9-P2O5
A1: 58-SiO, 32-CaO, 9-P2O5, 1-Ag2O
A1Z5: 58-SiO, 27-CaO, 9-P2O5, 1-Ag2O, 5-ZnO
Z5: 58-SiO, 28-CaO, 9-P2O5, 5-ZnO
