# Supplementary material for: Remineralization and anti-demineralization effect of orthodontic adhesives on enamel surrounding orthodontic brackets: a systematic review of in vitro studies
Source: BMC Oral Health. 2024 Nov 28;24:1446. doi: 10.1186/s12903-024-05237-y (PMC11603835; doi:10.1186/s12903-024-05237-y)
Supplement: Supplementary file 1 — Supplementary Material 1 [file 12903_2024_5237_MOESM1_ESM.docx]

**Supplementary file.1:** Search strategy in different databases

| **Database** | **Search keywords** |
| --- | --- |
| **PubMed** | ((((((((((((((Enamel) AND (Remineralization)) OR (Remineralisation))) AND (Orthodontic Adhesive)) OR (Orthodontic Adhesives)) OR (Bond)) AND (White spot lesion)) OR (White spot lesions)) OR (Artificial enamel lesion)) OR (Artificial enamel lesions)) OR (Artificial carious lesion)) OR (Artificial carious lesions)) OR (Demineralization)) AND (Orthodontic bracket) |
| **Web of science** | (((((((((((((ALL=(Enamel)) AND ALL=(Remineralization)) OR ALL=(Remineralisation)) AND ALL=(Orthodontic adhesive)) OR ALL=(Orthodontic adhesives)) OR ALL=(Bond)) AND ALL=(White spot lesion)) OR ALL=(White spot lesions)) OR ALL=(Artificial enamel lesion)) OR ALL=(Artificial enamel lesions)) OR ALL=(Artificial carious lesion)) OR ALL=(Artificial carious lesions)) OR ALL=(Demineralization)) AND ALL=(Orthodontic bracket) |
| **Scopus** | ALL (enamel AND remineralization OR remineralisation AND orthodontic AND adhesive OR orthodontic AND adhesives OR bond AND white AND spot AND lesion OR white AND spot AND lesions OR artificial AND enamel AND lesion OR artificial AND enamel AND lesions OR artificial AND carious AND lesion OR artificial AND carious AND lesions OR demineralization AND orthodontic AND bracket) |
