# Supplementary material for: Remineralization and anti-demineralization effect of orthodontic adhesives on enamel surrounding orthodontic brackets: a systematic review of in vitro studies
Source: BMC Oral Health. 2024 Nov 28;24:1446. doi: 10.1186/s12903-024-05237-y (PMC11603835; doi:10.1186/s12903-024-05237-y)
Supplement: Supplementary file 3 — Supplementary Material 3 [file 12903_2024_5237_MOESM3_ESM.docx]

**Supplementary file.2:** Remineralization results

| **Results** | **Adhesives used in the study** | **Remineralization results** |
| --- | --- | --- |
| **Choi, A. et al**  **2021** | Self-adhesive resin cement SAR (Ortho Connect Flow; GC Corp, Tokyo, Japan) containing MBN nano particles with different conc. 0,1,3,5 wt.% | Compared to the control group (SAR, 73.5 ± 9.3 μm), the SAR + 1, 3, and 5% MBN groups (105.7 ± 13.0, 109.2 ± 10.2, and 118.8 ± 1.1 μm) showed a significant difference. The remineralization length increased as the MBN content of the sample increased |
| **Choi, A. et al**  **2022** | Self-adhesive resin cement containing MPC and MPN with different conc.  1-SAR  2-SAR + 3% MPC  3-SAR + 5% MPC  4-SAR + 1% MBN + 3% MPC  5-SAR + 3% MBN + 3% MPC | The results were illustrated in a graph  Compared to the control (SAR, 96.1 ± 19.5 μm), all the groups showed a significant difference in remineralization length. The remineralization length increased as the MBN content of the sample increased |
| **Iijima, M. et al 2013** | 1- 4-META/MMA-TBB-based fluoride containing resin adhesive (Super-Bond/F3).  2- Super-Bond (SEP resin adhesive)  3- Transbond Plus, SEP resin adhesive  4- Fuji Ortho LC, RMGIC | There was no significant difference in hardness among the four specimen groups at depths of 51 µm–96 µm from the enamel surface, except at a depth of 66 µm (Super bond F3= 6.29± 1.03, Super bond = 6.17± 1.13, Transbond plus= 4.66 ± 1.85, Fuji ortho= 6.14± 0.75) p=0.003.  The values of hardness for Super-Bond and Transbond Plus were lower than those in the other two groups at depths of 11 µm–46 µm from the enamel surface, although in some locations the differences were not significant.  **At 11um distance:**  **Superbond** (4.15± 2.26), **Transbond plus** (3.78 ±2.78), **Super bond/F** (5.42±1.54), **Fuji Ortho** (5.99±0.78) (p=0.015)  **At 46um distance**:  **Superbond** (4.15± 2.26), **Transbond plus** (4.56±2.13), **Super bond/F** (4.80±2.04), **Fuji Ortho** (5.99± 0.59) (p=0.01)  For specimens bonded with Super-Bond/F3, the hardness values at most locations were similar to those in specimens bonded with Fuji Ortho LC.  Additionally, the reductions in hardness for Super-Bond/F3 and Fuji Ortho LC were lower than those for the other materials |
| **Kim, Y. M. et al 2018** | Transbond™ XT (TXT)  Charmfil™ Flow  Adhesive containing Silver or zinc doped bioactive glass BAG with different conc.  1-Charm Fill CF  2- Transbond TXT  3- CF+A0-10  4- CF+A1-10  5-CF+A1Z 5-10  6-CF+A1Z 5-15  7- CF+Z5-15 | BAG-containing orthodontic bonding agents Showed superior remineralized width  The greatest remineralization width was found in group Z5 (0.345± 0.02), while the lowest remineralization width was found TXT, CF control groups (0.048±0.02) (0.143±0.02) respectively.  There was significant difference between experimental (BAG-containing) and control adhesives (p=0.00) |
| **Kohda, N. et al**  **2015** | 4-META/ MMA-TBB-based resin adhesive with different conc. of BAG (0-50Wt.%) | There were no significant differences in hardness among the six specimen groups at most locations (depths) between depths of 21 and 48.5 μm from the enamel surface.  The values of hardness for specimens bonded with the 4-META/MMA-TBB-based resin with high BG content (40% and 50%) were higher than those in the other groups at some locations, between depths of 1 and 18.5 μm from the enamel surface |
| **Lee, S. M. et al 2017** | Sterile saline (-ve control)  1-Adhesive + BAG 0.2 wt%  2-Adhesive + BAG 1 wt%  3-Adhesive + BAG@Ag1 0.2%  4-Adhesive + BAG@Ag1 1%  5-Adhesive + BAG@Zn50.2%  6- Adhesive + BAG@Zn5 1% | The remineralization results were represented in a graph  1% BAG, BAG@Ag1, and BAG@Zn5 groups showed a significant difference compared to the control group  Among the samples, the orthodontic bonding primer containing 1% of BAG@Ag1 showed the highest remineralization property. |
| **Liu, Yan. et al 2018** | (1) TransBond XT -control TB  (2) PEHB + 5% MAEDB [designated as PD]  (3) PEHB + 5% MAE-DB + 40% NACP [designated as PND] | The results were presented in graphs  There were no significant differences among the groups in occlusal buccal, cervical, and lingual locations at 30, 120um depth.  Under the bracket at 30 um, PND group showed higher hardness compared to TB and PD (p < 0.05). While at 120 um there were no significant differences between the groups. |
| **Ma, Y. et al 2017** | 1-Transbond XT (TB)  2-RMGI (GC Ortho LC)  3-RMGI+MPC+DMAHDM  4-RMGI+MPC+DMAHDM+NACP. | Polarized light microscope findings:  The least LD was found in the group “RMGI+MPC+DMAHDM+NACP” for all the three distance from 50 to 150μm, 150 to 250 μm, and 250 to 350 μm, from adhesive margin which were 42.81 ± 17.05 μm, 47.72 ± 18.99 μm and 58.18 ± 21.34 μm, respectively.  The LD of “RMGI+MPC+DMAHDM” group was significantly lower than RMGI control at both 50-150 μm and 150-250 μm ranges (p < 0.05), but not significant at the 250-350 μm range (p = 0.847)  Cross sectional microhardness results:  At points 100, 200, 300um from the adhesive margin, “RMGI+MPC+DMAHDM+NACP” had significant higher hardness than group “RMGI+MPC+DMAHDM”, RMGI control and TB control (p < 0.05)  The enamel of RMGI control had lower hardness than “RMGI+MPC+DMAHDM” from 25 to 150 μm depths at 100 μm distance (p < 0.05) |
| **Manfred, L. et al 2013** | Transbond XT (control)  Orthodontic new adhesives contained BAG in varying percentages:  BAG62  BAG65  BAG81  BAG85 | **Comparison of significant decrease in microhardness between BAG groups and control Transbond XT at 3 different locations up to 125um**  Distance 100 25 All BAGs < TB  50 All BAGs< TB  75 All BAGs < TB  100 81,85 BAGs <TB  125 81 BAG < TB  Distance 200 25 All BAGs < TB  50 All BAGs< TB  75 All BAGs < TB  100 62,81,85 BAGs <TB  125 All BAGs < TB  Distance 300 25 All BAGs < TB  50 All BAGs< TB  75 81,85 BAGs < TB  100 62, 81,85 BAGs <TB  125 All BAGs < TB  At 25 and 50 µm deep at all distances from the bracket edge, all BAG-Bond adhesives showed significantly less reduction in hardness than Transbond-XT (*P* < .05).  **Comparison of significant change in microhardness bamong BAG groups at 3 different locations up to 125um**  Distance 100 25 81BAG < 62,65 BAG  50 81 BAG< 62,65 BAG  75 81 BAG < 62,65,85 BAGs  100 81 BAG <65 BAG  125 No sig. difference  Distance 200 25 81,85 BAGs < 65 BAG  50 81BAG < 65 BAG  75 No sig. difference  100 81BAGs <65 BAG  125 No significant difference  Distance 300 25 81 BAG < 65 BAG  50 No sig. difference  75 81BAG < 62,65 BAGs  100 81,85 BAGs <65 BAG  125 81BAG < 65 BAG  At 100 µm from the bracket edge and at depths of 25, 50, and 75 µm, 81BAG-Bond had less reduction in enamel microhardness than 62BAG-Bond and 65BAG-Bond. |
| **Nam, H. et al 2019** | 1-Charm Fill Flow (CF)  2-CF+ FGtBAG 1 Wt.%  3-CF+ FGtBAG 3 Wt.%  4-CF+ FGtBAG 5 Wt.% | The remineralization abilities of FGtBAG1 (41.9 ± 8.6 µg), FGtBAG3 (211.2 ± 32.3 µg), and FGtBAG5 (605.2 ± 126.8 µg) were significantly higher than that of CF (3.8 ± 0.0 µg).  The remineralization power increases with increase FGtBAG content |
| **Nam, H. et al 2019** | 1-Transbond XT Low Flow (LV)  2-LV+ FGtBAG 1 Wt.%  3-LV+ FGtBAG 3 Wt.%  4-LV+ FGtBAG 5 Wt.% | Anti-demineralization distances of FBAG1 (37.9 ± 7.7 μm), FBAG3 (50.4 ± 25.3 μm), and FBAG5 (229.3 ± 70.2 μm) were significantly larger than those of LV (14.7 ± 3.4 μm). |
| **Nascimento, P. et al 2017** | Adhesive containing antibacterial monomer [2 (Methacryloyloxy)ethyl] trimethylammonium chloride (MADQUAT; Sigma-Aldrich) with varying conc.  0, 5, 10 wt % | Neither the adhesive, (p=0.959) nor the cariogenic challenge. (P =0.759) affected the demineralization under the orthodontic brackets in contrast, around the brackets, both factors adhesive type , (P = 0.006) and cariogenic challenge (P < 0.001) affected the values of demineralization  **Under bracket**     \| MADQUAT \| control \| Cariogenic challenge \| \| --- \| --- \| --- \| \| 0% \| 1.446.9 ±900.1 \| 1.286.2 ±830.2 \| \| 5% \| 1.401.5 ±1455.7 \| 1.428.9 ±1.135.9 \| \| 10% \| 1.383.1 ±1.299.6 \| 1.215.0 ±936.1 \|   Around bracket   \| MADQUAT \| control \| Cariogenic challenge \| \| --- \| --- \| --- \| \| 0% \| 1.580.1 ±827.0^Ba^ \| 9.823.1 ±4.138.3^Aa^ \| \| 5% \| 1.626.6 ±1.078.3^Ba^ \| 7.876.5 ±3.969.1^Aa^ \| \| 10% \| 1.582.0 ±862.0^Aa^ \| 3.932.5 ±1.817.2^Ab^ \|   Distinct upper case letters for line indicate significant difference. |
| **Parihar, A. et al 2024** | 1-Transbond-XT Resin Adhesive + Transbond primer  2-BAG-Bond + (Transbond-XT Primer, 3M Unitek)  BAG-Bond without primer | SEM analysis shows that the prepared MBG particles possess spherical morphology with generally uniform size. Additionally, heterogenous surface consisting of random-sized particles with sharp edges and voids among them are shown |
| **Paschos, E. et al 2009** | 1-Transbond Plus SEP and Transbond XT  2-37% phosphoric acid, Pro Seal (Reliance Orthodontic Products, Itasca, Ill) and Transbond XT  3-Clearfil Protect Bond (Kuraray Medical, Okayama, Japan) and Transbond XT  4-37% phosphoric acid and Light Bond (Reliance Orthodontic Products)  5- Ortho Conditioner and Fuji Ortho LC | With the manual definition of lesion onset (onset 1) from the data from the mCT (, there were significant differences between the 5 groups according to the Ld and the mineral loss (DZ equivalent). By far, the least Ld was in group 5, the resin-modified glass ionomer cement Fuji Ortho LC.  mineral loss (DZ equivalent)  confirmed these results. The least amount of demineralization was found for bonding with Fuji Ortho LC  (group 5)  By using predetermined mineral loss and lesion depth (on set2) from micro CT data, there was no significant difference between the groups except in fuji ortho LC which showed the least Lesion depth and mineral loss  Lesion depth evaluated by the manual definition of lesion onset (onset 1) from the mCT data   \|  \| Transbond \| proseal \| Clearfil  protect \| Light bond \| Fuji ortho \| \| --- \| --- \| --- \| --- \| --- \| --- \| \| Mean±  SD \| 167.6± 18.8^C^ \| 156.0± 18.8^B^ \| 156.3± 16.3^B^ \| 150.3±16^B^ \| 126.5± 22.9^A^ \|   Mineral loss(DZ equivalent ) evaluated by the manual definition of lesion onset (onset 1) from the mCT data   \|  \| Transbond \| proseal \| Clearfil  protect \| Light bond \| Fuji ortho \| \| --- \| --- \| --- \| --- \| --- \| --- \| \| Mean±  SD \| 6987.9± 920.5^C^ \| 6611.6±  1012.9^BC^ \| 6407.6± 847.0^B^ \| 6181.6±  725.2^B^ \| 4907.4± 1304.1^A^ \|     lesion depth (Ld) evaluated by the predetermined lesion onset (onset 2) from the mCT data   \|  \| Transbond \| proseal \| Clearfil  protect \| Light bond \| Fuji ortho \| \| --- \| --- \| --- \| --- \| --- \| --- \| \| Mean±  SD \| 149.9± 17.6^B^ \| 141.9± 14.0^B^ \| 145.0± 14.4^B^ \| 137.4± 13.4^B^ \| 117.7± 21.1^A^ \|   mineral loss (DZ equivalent) evaluated by the predetermined lesion onset (onset 2) from the mCT data   \|  \| Transbond \| proseal \| Clearfil  protect \| Light bond \| Fuji ortho \| \| --- \| --- \| --- \| --- \| --- \| --- \| \| Mean±  SD \| 5945.4± 639.0^B^ \| 5855.5±  564.4^B^ \| 5744.2±  627.0^B^ \| 5547.8±  464.4^B^ \| 4302.3±  1172.8 ^A^ \|   Identical letters showed non-significant difference  Polarized light microscope findings are in agreement with micro CT findings as there was significant difference between Fuji Ortho LC and other tested materials (P=0.0001) The least mean (92.8 ± 23.5 )/ median (96.9±23.0) of lesion depth was found in Fuji Ortho LC . |
| **Rahmanpanah, S. et al 2023** | control group (3MTM Transbond TXT)  experimental composites containing 2% (HA2), 5% (HA5) and 10% (HA10) hydroxyapatite. | Micro hardness results presented in graph, HA5 and HA10 showed significantly higher microhardness compare to control group after demineralization process. The surface hardness of enamel in HA5 and HA10 groups after 4 weeks was significantly higher compared to control and demineralized samples (P < 0.05). HA10 showed the highest microhardness results  EDX, Results also presented in histogram illustration, the highest Ca, P wt.% was present in HA10.  SEM revealed that HA5, HA10 have different features compared to Control and HA2 groups as no course, deep demineralized enamel was detected but shallow mild demineralization along with nuclei of mineralization is perceptible |
| **Seifi, M. et al 2024** | 1- Experimental orthodontic composite (control).  2- Experimental orthodontic composite containing 1% nBG@Ag.  3-Experimental orthodontic composite containing 3% nBG@Ag.  4- Experimental orthodontic composite containing 5% nBG@Ag.  5- GC Ortho Connect orthodontic composite (control).  6- GC orthodontic composite containing 1% nBG@Ag.  7- GC orthodontic composite containing 3% nBG@Ag.  8- GC orthodontic composite containing 5% nBG@Ag. | one-way ANOVA results showed that the 8 examined groups before intervention did not have statistically significant differences (*p* = 0.753)  The secondary microhardness didn’t not have statistically significant differences in the 8 groups (*p* = 0.540)  SEM revealed that samples bonded with experimental composite containing 5% nBG@Ag showed porous enamel structures are covered with a layer of hydroxyapatite crystal deposits.  EDX findings revealed that samples bonded with 5% nBG@Ag showed calcium and phosphorus, with trace amounts of silica and silver |
| **Shirazi, M. et al 2019** | 1- control group: Transbond XT (3M, St. Paul, MN, USA)  2- Fuji II LC RMGI (GC Corp., Tokyo, Japan)  3- Fuji II LC (GC Corp., Tokyo, Japan) containing 30% BAG particles | **Lesion depth (Mean ± SD) by PLM**  BAG (73.8± 22.2)  RMGIC (118.08± 29.4)  Transbond XT (182.98± 20.6)  (*P*<0.0001)  The lowest lesion depth was reported in BAG group |
| **Song, H. et al 2019** | CharmFill Flow, Dentkist, Seoul, South Korea) containing 0, 1%, 3%, and 5% Ga-doped MBN (GaMBN) | Micro-CT findings:  As the concentration of GaMBN increased, the remineralization length increased (R = 0.76, p < 0.001)  Relative to GaMBN0 (53.7 ± 22.2 μm)  GaMBN1 (477.5 ± 260.5 μm)  GaMBN3 (728.4 ± 266.8 μm)  GaMBN5 (970.3 ± 370.9 μm)  P<0.001 |
| **Xu, Y. et al 2023** | 1-Transbond XT Primer (control)  2- 0 wt% AFCP  3- 25 wt% AFCP  4- 30 wt% AFCP | Color value (mean±SD) after 21 day  Control (14.64 ± 2.52)  0 wt% AFCP ( 16.52 ± 1.91)  25 wt% AFCP (10.63 ± 2.62)  30 wt % AFCP **(7.10 ± 1.05)**  Among the four groups, the 35 wt% AFCP group showed the least color alterations (P < 0.05).  Micro-CT findings:  After 3 weeks of pH cycling, the mean gray value profiles of the control and 0 wt% AFCP groups demonstrated a reduction in the gray value  25 wt% AFCP group displayed a reduced decrease in the gray value, while 35 wt% AFCP group showed the highest reduction of gray value, additionally 35 wt% AFCP group did not significantly vary after pH cycling  SEM revealed that , the porosities among enamel crystals were increased in the control and 0 wt% AFCP groups, while in the 25 wt% AFCP and 35 wt% AFCP groups, the original microstructure of enamel was well conserved and mineral deposits were clearly observed between enamel prisms |
| **Yi, J. et al 2019** | 1- Transbond XT (termed as TB control)  2- GC control  3- GC + 5% nCaF2  4- GC + 10% nCaF2  5- GC + 20% nCaF2  6- GC + 30% nCaF2  7- GC + 1% DMAHDM  8- GC + 2% DMAHDM  9- GC + 3% DMAHDM  10- GC + 4% DMAHDM  11- GC + 20% nCaF2 + 3% DMAHDM | The hardness results were presented in a plot  After the 30-day demineralization-remineralization cycles, the enamel hardness of TB control group was significantly decreased, compared to sound enamel (p < 0.05).  GC control and GC + 3% DMAHDM group showed greater enamel hardness than TB control (p < 0.05).  The two groups containing nCaF2 had even greater enamel hardness compared to TB control, GC control, and GC + 3% DMAHDM (p < 0.05).  The lesion depth was presented in a plot:  The TB control had the highest lesion depth (p < 0.05), followed by GC control and GC + 3% DMAHDM group.  GC + 20% nCaF2 group and GC + 20% nCaF2 + 3% DMAHDM group had the lowest lesion depths (p < 0.05) |
| **Bhushan, R. et al 2021** | 1-Transbond XT  2-Transbond Plus color change adhesive  3-GC Fuji Ortho LC  4- Vitremer | Comparison of mean enamel demineralization between control group and experimental groups after 14 days  Group I: Control (141.88 ± 1.09 )  Group II: Transbond Plus color change adhesive (108.19 ± 0.68)  Group III: GC Fuji Ortho LC (119.24 ± 0.37)  Group IV: Vitremer (121.56 ± 0.92)  (P=0.001)  Transbond Plus color change adhesive group was more power in inhibition of demineralization areas in comparison to GC Fuji Ortho LC group and Vitremer group |
| **Demircioglu, R. et al. 2023** | 1-Transbond XT Primer + Transbond XT Light Cure Adhesive (3M Unitek, Monrovia, CA, USA),  2-GC Ortho Connect Light Cure Adhesive (GC Crop, Tokyo, Japan)  3- Transbond™ Plus Self Etching Primer + Transbond XT Light Cure Adhesive (3M Unitek, Monrovia, CA, USA) | \|  \| TXT BOND+ primer (Median) \| GC ortho LC  (Median) \| TXT BOND PLUS+ primer  (Median) \| \| --- \| --- \| --- \| --- \| \| T0 \| 3.00 \| 3.00 \| 2.50 \| \| TI \| 6.00 \| 7.00 \| 5.00 \| \| T1-T0 \| 4.00 \| 4.00 \| **3.00** \|   **DIAGNODent findings:**  All the groups showed statistically significant increase of demineralization around brackets gingivally, proximally, and occlusally.  Only in Occlusal assessment TXT Bond plus+ primer showed significant reduction in demineralization compared to TXT BOND+ Primer and GC ortho Lc |
| **Chow, C. et al 2011** | (1) Transbond XT (3M Unitek, Monrovia, CA), a light cure composite without fluoride or ACP.  (2) Quick Cure (Reliance Orthodontic Products, Itasca, IL), a light cure composite with fluoride.  (3) Aegis Ortho (Bosworth Co., Skokie, IL), a light cure composite with 38% ACP fillers | **XPS findings**  The Kruskal Wallis Test revealed that Transbond XT, Quick Cure, and Aegis Ortho had significant differences in elemental concentration; Ca (*P* = 0.032), P (*P* = 0.002), Si (*P* = 0.028), C (*P* = 0.019).  By using Mann whiteney test to test the relation between the different groups, there were no significant differences in elemental concentrations between Transbond XT and Quick Cure. There were significant differences between Transbond XT and Aegis Ortho; Ca (*P* = 0.007), P (*P* = 0.001), Si (*P* = 0.038), C (*P* = 0.004) as calcium decreased at the end of the examination period in Aegis Ortho (T2, 16.46%) compared to Transbond XT (T2, 24.08%)  **PLM Findings:**  Control (155 ± 23 *μ*m)  Transbond XT (161 ± 38 *μ*m)  Quick Cure (117 ± 26 *μ*m)  Aegis Ortho ( 123 ± 31 *μ*m)  Kruskal-Wallis testing showed significance differences between the groups (*P* = 0.02). Quick Cure showed the highest reduction in lesion depth. |
| **Firoz, H. et al. 2019** | 1-F-BGC-1: Fluoride bioactive glass ceramic + Transbond XT B.  2- BGC-1: Bioactive glass ceramic + Transbond XT C.  3- F-BGC-2 Fluoride bioactive glass ceramic + Transbond XT  4- BGC-2 Bioactive glass ceramic + Transbond XT.  5- Control Transbond XT only | Experimental groups depicted large remineralization peaks at 1001-1005 cm−1 and smaller peaks of remineralization were due to C=H, C=O, and C=C, respectively from the polymeric network of Transbond XT compared to control group.  SEM. The radiopaque particles of the BGC adhesives were seen scattered over the surface in group 2 and 3.  Among the entire images, group 3 exhibited the highest organization pattern of enamel structure where the rods were distinct and the BGC adhesive was evident. |

**Abbreviations:**

**MPC**; 2-methacryloyloxyethyl phosphorylcholine. **nHAP**: nano-hydroxy appetite. **nACP**: nano-hydroxyapatite. **CSMH**: Cross sectional microhardness

**CSNH**: cross sectional nano hardness. **Micro-CT**: micro-computed tomography **PLM**: Polarized light microscope **SEM**: Scan Electron Microscope

**XRD**: X-Ray Diffraction **EDX**: Energy Dispersing X-ray **AFM:** Atomic Force Microscope. **nCaF2:** nano Calcium-flouride. **BAG**: Bioactive Glass

**AFCP:** Amorphous Fluorinated Calcium Phosphate nanoparticles. **(MAE-DB):** 2-methacryloxylethyl dodecyl methyl ammonium bromide

**MPN:** Mesoporous Bioactive glass nanoparticles. **DMAHDM**: di-methylamino hexadecyl methacrylate. **nBG@Ag:** nano-silver containing bioactive glass

**FGtBAG:** fluorinated bioactive glass nanoparticles. **PEHB:** orthodontic experimental adhesive containing [PMGDM, EBPADMA, HEMA, Bis-GMA, BAPO]

**PD**: PEHB + 5% MAEDB. **PND:** PEHB + 5% MAE-DB + 40% NACP. **SAR:** Self -adhesive resin cement, **META/ MMA-TBB**: 4-Acryloyloxyethyl trimellitate anhydride/methyl methacrylate-tri-n-butylborane. **SEP:** self-etch primer. **MBN:** Mesoporous Bioactive glass Nanoparticles. **F-BGC**: Flouridated bioglass ceramic based adhesive. **GaMBN:** Gallium-doped bioactive glass nanoparticles. **A0**: 58-SiO, 33-CaO, 9-P2O5. **A1:** 58-SiO, 32-CaO, 9-P2O5, 1-Ag2O

**A1Z5:** 58-SiO, 27-CaO, 9-P2O5, 1-Ag2O, 5-ZnO. **Z5:** 58-SiO, 28-CaO, 9-P2O5, 5-ZnO
