# Supplementary material for: Remineralization and anti-demineralization effect of orthodontic adhesives on enamel surrounding orthodontic brackets: a systematic review of in vitro studies
Source: BMC Oral Health. 2024 Nov 28;24:1446. doi: 10.1186/s12903-024-05237-y (PMC11603835; doi:10.1186/s12903-024-05237-y)
Supplement: Supplementary file 4 — Supplementary Material 4 [file 12903_2024_5237_MOESM4_ESM.docx]

**Supplementary file.3:** Ion release results

|  | **Ion release results** | **Summary of findings** |
| --- | --- | --- |
| Iijima, M. et al 2013 | Total fluoride Ion release.   \|  \| **Super bond /F3** \| **Super Bond** \| **Transbond plus** \| **Fuji Ortho LC** \| \| --- \| --- \| --- \| --- \| --- \| \| **1 day** \| 20.55± 6.26^a^ \| 0.49±  0.27^b^ \| 2.32±  0.23^c^ \| 9.01±  0.93^d^ \| \| **3days** \| 29.85± 8.80^a^ \| 0.86± 0.36^b^ \| 3.95±  0.34^c^ \| 25.55±  1.28^a^ \| \| **7days** \| 38.00± 10.48^a^ \| 0.95± 0.46^b^ \| 6.12±  0.34^c^ \| 37.35±  1.83^a^ \| \| **14 days** \| 46.13± 11.68^a^ \| 0.99 ±0.47^b^ \| 7.99± 0.35^c^ \| 47.78±  1.76^a^ \| \| **28 days** \| 49.92±12.44 \| 1.04± 0.46^b^ \| 10.11± 0.43^c^ \| 57.53±  2.54^a^ \| \| **6 months** \| 65.27± 12.87^a^ \| 1.09± 0.46^b^ \| 19.99± 0.88^c^ \| 104.98±  3.00^d^ \|   Identical letters mean non statistical significance. | The amount of fluoride ions released from Super-Bond was below the detection limit  Fuji Ortho LC (104.98 ppm) showed significantly greater total fluoride release compared with the other groups during the 6-month period. |
| Kim, Y. M. et al 2018 | _ | Ion release was examined for 1, 6, 24, 72, 168, or 840 hours. Calcium and phosphate ions concentration decreased in all samples after 72 hours.  Silver  A1-10 and A1Z5-10 groups and its concentration increased up to 840 hour, additionally zinc was detected up to 840 hour in A1Z5-10 group.  No pH changes were observed with the passage of time |
| Kohda, N. et al  2015 | _ | After the first day of immersion, Ca, Na, Si, and B were detected in solutions with BG-containing 4-META/MMA-TBB-based resin specimens. All ions (Ca, Na, Si, and B) were continuously released during an immersion period of 3 months and the amounts of ions released during this period increased with increasing BG content.  Acid neutralizing and PH increased with increasing BG content |
| Liu, Yan. et al 2018 | Concentration of Ca and P ions after 42 days in three adhesives   \|  \| **PND** \| **PD** \| **TB** \| \| --- \| --- \| --- \| --- \| \| **Ca** \| 80.16 ± 2.21 mg/L \| 0.60 ± 0.09 mg/L \| 0.51 ± 0.06 mg/L \| \| **P** \| 50.16 ± 1.46 mg/L \| 0.068 ± 0.04 mg/l \| Below minimum detectable limit \| | The Ca and P ions released from the TB and PD groups approximated null values up to a period of 42 days. In contrast, PND showed high levels of ion release. |
| Nam, H. et al 2019 | FGtBAG1 showed fluorine ion release of 6.9–10.1 µg/cm^2^ from 12 hour to 20 days. FGtBAG3 and FGtBAG5 showed fluorine ion release of 9.2–16.7 µg/cm^2^ and 9.1–17.3 µg/cm^2^, respectively | As the FGt content increased, the fluorine ion release rate also increased. |
| Nam, H. et al 2019 | After a period between 12 hour and 20 days, the fluoride ion release was 11.1–17.4 μg/cm^2^ for FBAG1, 13.2–14.91 μg/cm^2^ for FBAG3, and 9.5–15.6 μg/cm^2^ for FBAG5. | No significant differences in fluoride ions release were detected between the 3 groups. |
| Song, H. et al 2019 | P ion release in **GaMBN1:**  1 day (0.4 ±00ppm)  7 days (1.2±00ppm)  14 days (0.8±00ppm)  P ion release for **GaMBN3:**  1 day (1.00 ±00ppm)  7 days (3.3±01ppm)  14 days (3.4±01ppm)  P ion release for **GaMBN5**:  1 day (1.3 ±01ppm)  7 days (4.5±01ppm)  14 days (6.4±01ppm)  No P ion released with **GaMBN 0**  The pH decreased with increasing GaMBN content, and generally decreased with immersion time for each disk | Concentration of P ion release increased with increasing GaMBN Content |
| Yi, J. et al 2019 | _ | The amount of released F ions was significantly enhanced up to 56 days by incorporating nCaF2 and reduction of PH value (p < 0.05), while the incorporation of DMAHDM did not affect the F release (p > 0.1)  The concentration of Ca ions releases up to 56 days increased with incorporation of nCaF2 and reduction of PH (p > 0.1). Calcium ion release was significantly higher in the two groups containing nCaF2 than GC control group and GC + 3% DMAHDM group up to 56 days (p < 0.05). |

**FGt**: fluorinated graphite

**PEHB:** orthodontic experimental adhesive containing [PMGDM, EBPADMA, HEMA, Bis-GMA, BAPO]

**PD**: PEHB + 5% MAEDB

**PND:** PEHB + 5% MAE-DB + 40% NACP

**META/ MMA-TBB**: 4-Acryloyloxyethyl trimellitate anhydride/methyl methacrylate-tri-n-butylborane

**GaMBN:** Gallium-doped bioactive glass nanoparticles

**nCaF2:** nano Calcium-flouride.

**DMAHDM**: di-methylamino hexadecyl methacrylate

**A0**: 58-SiO, 33-CaO, 9-P2O5.

**A1:** 58-SiO, 32-CaO, 9-P2O5, 1-Ag2O

**A1Z5:** 58-SiO, 27-CaO, 9-P2O5, 1-Ag2O, 5-ZnO.

**Z5:** 58-SiO, 28-CaO, 9-P2O5, 5-ZnO
